# *tert*-Butyl 3-benzyl-3-[(*E*)-2-benzyl­idene-3-oxocyclo­pent­yl]-2-oxoindoline-1-carboxyl­ate

**DOI:** 10.1107/S160053681100691X

**Published:** 2011-03-12

**Authors:** Zhen Qiao, Li Liu, Dong Wang

**Affiliations:** a Beijing National Laboratory for Molecular Science (BNLMS), CAS Key Laboratory for Molecular Recognition and Function, Institute of Chemistry, Chinese Academy of Sciences, Beijing 100190, People’s Republic of China

## Abstract

In the title compound, C_32_H_31_NO_4_, the dihedral angles between the indoline ring and the two phenyl rings are 48.11 (9) and 66.55 (9)°. The mol­ecular conformation is stabilized by a weak intramolecular π–π stacking inter­action [centroid–centroid distance = 3.6377 (7) Å]. The crystal structure is stabilized by inter­molecular C—H⋯O hydrogen bonds, which form chains along the *b* axis.

## Related literature

For the preparation of chiral 3,3-disubstituted 2-oxindoles, see: Cozzi *et al.* (2009 ▶); Qiao *et al.* (2010 ▶); Zhou *et al.* (2010 ▶). For bond-length data, see: Allen *et al.* (1987 ▶).
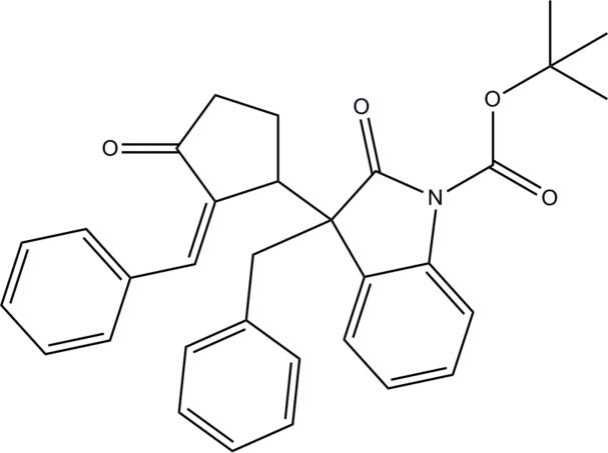


## Experimental

### 

#### Crystal data

C_32_H_31_NO_4_
*M* *_r_* = 493.58
Orthorhombic, 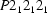

*a* = 7.8695 (16) Å
*b* = 11.893 (2) Å
*c* = 28.346 (6) Å
*V* = 2652.9 (9) Å^3^
*Z* = 4
Mo *K*α radiation
μ = 0.08 mm^−1^
*T* = 173 K
0.35 × 0.26 × 0.12 mm

#### Data collection

Rigaku Saturn724+ diffractometer
Absorption correction: multi-scan (*CrystalClear*; Rigaku, 2008 ▶) *T*_min_ = 0.525, *T*_max_ = 1.000
9151 measured reflections
2967 independent reflections
2651 reflections with *I* > 2σ(*I*)
*R*_int_ = 0.088

#### Refinement

*R*[*F* ^2^ > 2σ(*F* ^2^)] = 0.055
*wR*(*F* ^2^) = 0.132
*S* = 1.05
2967 reflections
334 parameters
H-atom parameters constrained
Δρ_max_ = 0.20 e Å^−3^
Δρ_min_ = −0.23 e Å^−3^

### 

Data collection: *CrystalClear* (Rigaku, 2008 ▶); cell refinement: *CrystalClear*; data reduction: *CrystalClear*; program(s) used to solve structure: *SHELXS97* (Sheldrick, 2008 ▶); program(s) used to refine structure: *SHELXL97* (Sheldrick, 2008 ▶); molecular graphics: *SHELTXL* (Sheldrick, 2008 ▶); software used to prepare material for publication: *SHELXTL*.

## Supplementary Material

Crystal structure: contains datablocks I, global. DOI: 10.1107/S160053681100691X/rz2560sup1.cif

Structure factors: contains datablocks I. DOI: 10.1107/S160053681100691X/rz2560Isup2.hkl

Additional supplementary materials:  crystallographic information; 3D view; checkCIF report

## Figures and Tables

**Table 1:** Hydrogen-bond geometry (Å, °)

*D*—H⋯*A*	*D*—H	H⋯*A*	*D*⋯*A*	*D*—H⋯*A*
C21—H21*A*⋯O4^i^	1.00	2.37	3.295 (4)	153
C28—H28*A*⋯O4^i^	0.95	2.47	3.401 (4)	165

Symmetry code: (i) 
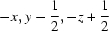
.

**Fig. 1.**
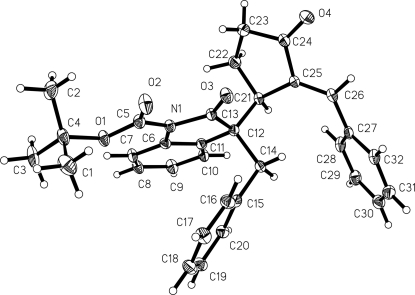
The molecular structure of title compound, with atom labels and 30% probability displacement ellipsoids for non-H atoms.

## supplementary crystallographic information

### Comment

Recently, as important substructures, chiral 3,3-disubstituted 2-oxindoles have constituted a ubiquitous class of heterocycles found in numerous natural products, marketed drugs, and drug candidates. Though a handful of synthetic methods are available for creating the single quaternary carbon centers at the C-3 position with complete control, the challenge still lies primarily in the efficient construction of a vicinal chiral tertiary carbon centre. Meanwhile, the direct catalytic asymmetric substitution of alcohols represents a highly challenging and persistent problem for synthetic methodology. Because of the inert leaving ability of the hydroxyl group, the catalytic activation of alcohols is generally difficult (Cozzi *et al.*, 2009). By using our method we can overcome the problem mentioned above (Qiao *et al.*, 2010; Zhou *et al.*, 2010). We report here the crystal structure of the title compound.

In title compound (Fig. 1), all bond lengths are normal (Allen *et al.*, 1987). The dihedral angle between the indoline (C6—C13/N1) and the C15—C20 and C27—C32 phenyl rings are 48.11 (9) and 66.55 (9) °, respectively. A weak intramolecular π—π interaction is indicated by the distance of 3.6377 (7) Å between the centroids of the pyrrole ring (N1/C6/C11–13) and C15—C20 phenyl ring. In the crystal structure, weak C—H···O intermolecular hydrogen interactions link molecules into chains along the *b* axis (Table 1).

### Experimental

To a solution of catalyst salt 9-amino-9-deoxyepiquinine (20 mol %) in the combination with TFA (40 mol %) in 1 ml DCM, 5 min s later, the Baylis-Hillman adduct 2-(hydroxy(phenyl)methyl)cyclopent-2-enone (0.2 mmol) and *tert*-butyl 3-benzyl-2-oxoindoline-1-carboxylate (0.2 mmol) was added. The resulting reaction mixture was heated at 30° C and stirred for 96 h till completion as judged by TLC. Then the reaction mixture was purified by column chromatography over silica gel (gradient petroleum ether/EtOAc 6:1 *v*/*v*) to afford the title compound in 32% yield. Single crystals suitable for X-ray measurements were obtained by recrystallization from acetonitrile at room temperature.

### Refinement

H atoms were positioned geometrically and refined using a riding model, with C—H = 0.95–0.99 Å and with *U*_iso_(H) = 1.2 *U*_eq_(C) for CH and CH2 or 1.5 *U*_eq_(C) for methyl H atoms. In the absence of anomalous dispersion effects, Friedel pairs were merged before the final cycles of refinement.

### Crystal data

**Table:**

C_32_H_31_NO_4_	*F*(000) = 1048
*M**_r_* = 493.58	*D*_x_ = 1.236 Mg m^−^^3^
Orthorhombic, *P*2_1_2_1_2_1_	Mo *K*α radiation, λ = 0.71073 Å
Hall symbol: P 2ac 2ab	Cell parameters from 7550 reflections
*a* = 7.8695 (16) Å	θ = 1.4–26.0°
*b* = 11.893 (2) Å	µ = 0.08 mm^−^^1^
*c* = 28.346 (6) Å	*T* = 173 K
*V* = 2652.9 (9) Å^3^	Block, colorless
*Z* = 4	0.35 × 0.26 × 0.12 mm

### Data collection

**Table:**

Rigaku Saturn724+ diffractometer	2967 independent reflections
Radiation source: Rotating Anode	2651 reflections with *I* > 2σ(*I*)
Confocal	*R*_int_ = 0.088
Detector resolution: 28.5714 pixels mm^-1^	θ_max_ = 26.0°, θ_min_ = 1.9°
ω scans at fixed χ = 45°	*h* = −9→6
Absorption correction: multi-scan (*CrystalClear*; Rigaku, 2008)	*k* = −14→14
*T*_min_ = 0.525, *T*_max_ = 1.000	*l* = −18→34
9151 measured reflections	

### Refinement

**Table:**

Refinement on *F*^2^	Primary atom site location: structure-invariant direct methods
Least-squares matrix: full	Secondary atom site location: difference Fourier map
*R*[*F*^2^ > 2σ(*F*^2^)] = 0.055	Hydrogen site location: inferred from neighbouring sites
*wR*(*F*^2^) = 0.132	H-atom parameters constrained
*S* = 1.05	*w* = 1/[σ^2^(*F*_o_^2^) + (0.0628*P*)^2^ + 0.4619*P*] where *P* = (*F*_o_^2^ + 2*F*_c_^2^)/3
2967 reflections	(Δ/σ)_max_ < 0.001
334 parameters	Δρ_max_ = 0.20 e Å^−^^3^
0 restraints	Δρ_min_ = −0.23 e Å^−^^3^

### Special details

**Table:**

Geometry. All e.s.d.'s (except the e.s.d. in the dihedral angle between two l.s. planes) are estimated using the full covariance matrix. The cell e.s.d.'s are taken into account individually in the estimation of e.s.d.'s in distances, angles and torsion angles; correlations between e.s.d.'s in cell parameters are only used when they are defined by crystal symmetry. An approximate (isotropic) treatment of cell e.s.d.'s is used for estimating e.s.d.'s involving l.s. planes.
Refinement. Refinement of *F*^2^ against ALL reflections. The weighted *R*-factor *wR* and goodness of fit *S* are based on *F*^2^, conventional *R*-factors *R* are based on *F*, with *F* set to zero for negative *F*^2^. The threshold expression of *F*^2^ > σ(*F*^2^) is used only for calculating *R*-factors(gt) *etc*. and is not relevant to the choice of reflections for refinement. *R*-factors based on *F*^2^ are statistically about twice as large as those based on *F*, and *R*- factors based on ALL data will be even larger.

### Fractional atomic coordinates and isotropic or equivalent isotropic displacement parameters (Å^2^)

**Table:**

	*x*	*y*	*z*	*U*_iso_*/*U*_eq_	
O1	0.1446 (3)	0.51425 (19)	0.50716 (6)	0.0331 (5)	
O2	0.1567 (4)	0.6816 (2)	0.46945 (7)	0.0458 (7)	
O3	0.1853 (3)	0.66366 (19)	0.37455 (7)	0.0341 (5)	
O4	−0.0170 (4)	0.7532 (2)	0.26192 (8)	0.0484 (7)	
N1	0.1590 (3)	0.5157 (2)	0.42829 (7)	0.0247 (5)	
C1	0.2941 (6)	0.6257 (4)	0.56693 (13)	0.0553 (11)	
H1A	0.3020	0.6934	0.5474	0.083*	
H1B	0.3916	0.5767	0.5605	0.083*	
H1C	0.2941	0.6471	0.6003	0.083*	
C2	−0.0256 (6)	0.6350 (4)	0.55892 (11)	0.0488 (10)	
H2A	−0.0119	0.7023	0.5393	0.073*	
H2B	−0.0437	0.6574	0.5918	0.073*	
H2C	−0.1238	0.5918	0.5478	0.073*	
C3	0.1162 (7)	0.4580 (3)	0.58547 (10)	0.0559 (12)	
H3A	0.0102	0.4189	0.5776	0.084*	
H3B	0.1154	0.4786	0.6189	0.084*	
H3C	0.2129	0.4083	0.5791	0.084*	
C4	0.1317 (5)	0.5638 (3)	0.55551 (9)	0.0381 (9)	
C5	0.1538 (4)	0.5817 (3)	0.46984 (9)	0.0302 (7)	
C6	0.1527 (4)	0.3956 (3)	0.42408 (9)	0.0248 (6)	
C7	0.1196 (4)	0.3132 (3)	0.45733 (9)	0.0316 (7)	
H7A	0.1044	0.3320	0.4896	0.038*	
C8	0.1092 (5)	0.2022 (3)	0.44210 (10)	0.0335 (8)	
H8A	0.0869	0.1449	0.4646	0.040*	
C9	0.1304 (5)	0.1729 (3)	0.39534 (10)	0.0368 (8)	
H9A	0.1213	0.0965	0.3858	0.044*	
C10	0.1651 (5)	0.2567 (3)	0.36226 (9)	0.0291 (7)	
H10A	0.1804	0.2379	0.3300	0.035*	
C11	0.1771 (4)	0.3667 (3)	0.37675 (9)	0.0252 (6)	
C12	0.2152 (4)	0.4694 (3)	0.34814 (9)	0.0241 (6)	
C13	0.1835 (4)	0.5643 (3)	0.38325 (9)	0.0250 (6)	
C14	0.4084 (4)	0.4752 (3)	0.33532 (9)	0.0262 (6)	
H14A	0.4353	0.4136	0.3131	0.031*	
H14B	0.4312	0.5472	0.3190	0.031*	
C15	0.5246 (4)	0.4662 (3)	0.37767 (9)	0.0270 (6)	
C16	0.5679 (5)	0.5611 (3)	0.40327 (11)	0.0352 (8)	
H16A	0.5254	0.6324	0.3939	0.042*	
C17	0.6733 (5)	0.5529 (3)	0.44273 (11)	0.0384 (8)	
H17A	0.7012	0.6183	0.4603	0.046*	
C18	0.7361 (5)	0.4508 (3)	0.45593 (11)	0.0376 (8)	
H18A	0.8081	0.4454	0.4827	0.045*	
C19	0.6955 (5)	0.3543 (3)	0.43046 (11)	0.0370 (8)	
H19A	0.7404	0.2834	0.4395	0.044*	
C20	0.5884 (4)	0.3630 (3)	0.39157 (10)	0.0303 (7)	
H20A	0.5588	0.2973	0.3744	0.036*	
C21	0.1013 (4)	0.4759 (3)	0.30321 (8)	0.0249 (6)	
H21A	0.1178	0.4061	0.2841	0.030*	
C22	−0.0884 (4)	0.4869 (3)	0.31628 (11)	0.0350 (8)	
H22A	−0.1590	0.4424	0.2942	0.042*	
H22B	−0.1081	0.4587	0.3487	0.042*	
C23	−0.1352 (4)	0.6112 (3)	0.31313 (11)	0.0365 (8)	
H23A	−0.2516	0.6207	0.3005	0.044*	
H23B	−0.1289	0.6473	0.3446	0.044*	
C24	−0.0066 (4)	0.6608 (3)	0.28029 (9)	0.0332 (7)	
C25	0.1313 (4)	0.5785 (3)	0.27179 (9)	0.0247 (6)	
C26	0.2475 (4)	0.5990 (3)	0.23824 (9)	0.0292 (7)	
H26A	0.2390	0.6701	0.2231	0.035*	
C27	0.3871 (4)	0.5258 (3)	0.22173 (9)	0.0272 (7)	
C28	0.3654 (5)	0.4114 (3)	0.21275 (9)	0.0341 (8)	
H28A	0.2567	0.3780	0.2167	0.041*	
C29	0.5013 (5)	0.3463 (3)	0.19813 (10)	0.0377 (8)	
H29A	0.4844	0.2687	0.1918	0.045*	
C30	0.6608 (5)	0.3924 (3)	0.19263 (10)	0.0380 (8)	
H30A	0.7540	0.3469	0.1832	0.046*	
C31	0.6829 (5)	0.5059 (3)	0.20108 (10)	0.0370 (8)	
H31A	0.7923	0.5386	0.1974	0.044*	
C32	0.5480 (4)	0.5724 (3)	0.21471 (9)	0.0322 (8)	
H32A	0.5647	0.6507	0.2194	0.039*	

### Atomic displacement parameters (Å^2^)

**Table:**

	*U*^11^	*U*^22^	*U*^33^	*U*^12^	*U*^13^	*U*^23^
O1	0.0538 (14)	0.0263 (12)	0.0191 (8)	0.0018 (12)	0.0009 (9)	−0.0037 (8)
O2	0.082 (2)	0.0251 (14)	0.0307 (10)	−0.0009 (14)	0.0073 (12)	−0.0026 (9)
O3	0.0499 (15)	0.0230 (12)	0.0293 (9)	−0.0013 (11)	0.0010 (10)	0.0023 (9)
O4	0.0520 (16)	0.0397 (16)	0.0535 (14)	0.0141 (14)	0.0082 (13)	0.0196 (12)
N1	0.0321 (14)	0.0203 (13)	0.0218 (10)	−0.0001 (12)	0.0004 (10)	0.0006 (9)
C1	0.076 (3)	0.048 (3)	0.0428 (18)	−0.004 (2)	−0.0177 (19)	−0.0079 (18)
C2	0.068 (3)	0.045 (2)	0.0335 (15)	0.007 (2)	0.0099 (16)	−0.0113 (16)
C3	0.110 (4)	0.036 (2)	0.0212 (14)	0.001 (3)	0.0062 (18)	−0.0010 (14)
C4	0.064 (2)	0.0310 (18)	0.0191 (12)	0.0043 (19)	−0.0019 (14)	−0.0052 (12)
C5	0.0381 (19)	0.0288 (19)	0.0237 (13)	0.0013 (15)	0.0017 (13)	−0.0013 (11)
C6	0.0274 (16)	0.0228 (16)	0.0242 (12)	0.0024 (14)	−0.0005 (12)	0.0012 (11)
C7	0.043 (2)	0.0315 (18)	0.0207 (12)	−0.0003 (16)	0.0026 (12)	0.0037 (11)
C8	0.049 (2)	0.0223 (16)	0.0293 (13)	−0.0013 (17)	−0.0012 (14)	0.0100 (12)
C9	0.054 (2)	0.0247 (18)	0.0318 (14)	−0.0017 (17)	−0.0003 (15)	0.0000 (13)
C10	0.0405 (19)	0.0249 (16)	0.0221 (12)	0.0023 (15)	0.0026 (13)	0.0004 (11)
C11	0.0259 (16)	0.0255 (17)	0.0242 (12)	−0.0002 (14)	−0.0001 (12)	0.0014 (11)
C12	0.0260 (15)	0.0254 (17)	0.0210 (12)	0.0008 (13)	0.0015 (11)	0.0004 (11)
C13	0.0290 (16)	0.0248 (17)	0.0213 (12)	0.0002 (14)	0.0009 (11)	0.0020 (11)
C14	0.0290 (15)	0.0266 (16)	0.0231 (11)	0.0021 (14)	0.0004 (11)	0.0035 (11)
C15	0.0267 (15)	0.0293 (17)	0.0249 (12)	−0.0001 (14)	0.0054 (11)	0.0031 (12)
C16	0.0367 (19)	0.0313 (19)	0.0376 (15)	0.0008 (16)	−0.0026 (14)	0.0036 (14)
C17	0.042 (2)	0.036 (2)	0.0379 (16)	−0.0098 (18)	−0.0070 (15)	−0.0027 (14)
C18	0.0364 (19)	0.042 (2)	0.0346 (15)	−0.0053 (17)	−0.0077 (14)	0.0086 (14)
C19	0.0358 (19)	0.033 (2)	0.0417 (16)	−0.0011 (17)	−0.0043 (14)	0.0122 (14)
C20	0.0325 (17)	0.0247 (17)	0.0337 (14)	−0.0007 (15)	−0.0009 (13)	0.0019 (12)
C21	0.0305 (16)	0.0230 (16)	0.0211 (11)	−0.0020 (14)	0.0011 (11)	0.0021 (10)
C22	0.0321 (17)	0.040 (2)	0.0333 (13)	−0.0024 (16)	−0.0023 (13)	0.0053 (14)
C23	0.0325 (19)	0.040 (2)	0.0367 (14)	0.0036 (17)	0.0016 (13)	0.0073 (14)
C24	0.0347 (18)	0.038 (2)	0.0263 (12)	0.0012 (17)	−0.0016 (12)	0.0075 (13)
C25	0.0288 (16)	0.0253 (16)	0.0200 (11)	−0.0006 (13)	−0.0015 (11)	0.0015 (10)
C26	0.0380 (18)	0.0260 (17)	0.0237 (12)	−0.0022 (15)	−0.0013 (12)	0.0045 (11)
C27	0.0352 (17)	0.0280 (17)	0.0184 (11)	0.0012 (15)	0.0039 (11)	0.0051 (11)
C28	0.047 (2)	0.0288 (18)	0.0267 (14)	−0.0082 (17)	0.0071 (14)	0.0011 (12)
C29	0.052 (2)	0.031 (2)	0.0301 (13)	−0.0011 (18)	0.0061 (15)	−0.0014 (13)
C30	0.046 (2)	0.039 (2)	0.0292 (14)	0.0069 (18)	0.0035 (14)	0.0000 (14)
C31	0.0362 (18)	0.042 (2)	0.0325 (14)	−0.0014 (17)	0.0020 (13)	0.0014 (14)
C32	0.043 (2)	0.0274 (18)	0.0257 (13)	−0.0058 (15)	0.0040 (13)	0.0018 (11)

### Geometric parameters (Å, °)

**Table:**

O1—C5	1.330 (4)	C14—H14B	0.9900
O1—C4	1.495 (3)	C15—C20	1.383 (4)
O2—C5	1.188 (4)	C15—C16	1.384 (5)
O3—C13	1.207 (4)	C16—C17	1.396 (4)
O4—C24	1.219 (4)	C16—H16A	0.9500
N1—C13	1.415 (3)	C17—C18	1.363 (5)
N1—C5	1.416 (4)	C17—H17A	0.9500
N1—C6	1.434 (4)	C18—C19	1.393 (5)
C1—C4	1.510 (6)	C18—H18A	0.9500
C1—H1A	0.9800	C19—C20	1.391 (4)
C1—H1B	0.9800	C19—H19A	0.9500
C1—H1C	0.9800	C20—H20A	0.9500
C2—C4	1.503 (6)	C21—C25	1.529 (4)
C2—H2A	0.9800	C21—C22	1.544 (5)
C2—H2B	0.9800	C21—H21A	1.0000
C2—H2C	0.9800	C22—C23	1.526 (5)
C3—C4	1.523 (5)	C22—H22A	0.9900
C3—H3A	0.9800	C22—H22B	0.9900
C3—H3B	0.9800	C23—C24	1.496 (5)
C3—H3C	0.9800	C23—H23A	0.9900
C6—C7	1.384 (4)	C23—H23B	0.9900
C6—C11	1.398 (4)	C24—C25	1.481 (5)
C7—C8	1.391 (5)	C25—C26	1.342 (4)
C7—H7A	0.9500	C26—C27	1.478 (4)
C8—C9	1.381 (4)	C26—H26A	0.9500
C8—H8A	0.9500	C27—C28	1.395 (4)
C9—C10	1.396 (4)	C27—C32	1.396 (5)
C9—H9A	0.9500	C28—C29	1.384 (5)
C10—C11	1.375 (4)	C28—H28A	0.9500
C10—H10A	0.9500	C29—C30	1.379 (5)
C11—C12	1.496 (4)	C29—H29A	0.9500
C12—C13	1.526 (4)	C30—C31	1.382 (5)
C12—C21	1.560 (4)	C30—H30A	0.9500
C12—C14	1.564 (4)	C31—C32	1.379 (5)
C14—C15	1.513 (4)	C31—H31A	0.9500
C14—H14A	0.9900	C32—H32A	0.9500
			
C5—O1—C4	119.7 (2)	C20—C15—C16	119.0 (3)
C13—N1—C5	121.9 (3)	C20—C15—C14	120.6 (3)
C13—N1—C6	109.7 (2)	C16—C15—C14	120.4 (3)
C5—N1—C6	128.3 (2)	C15—C16—C17	120.6 (3)
C4—C1—H1A	109.5	C15—C16—H16A	119.7
C4—C1—H1B	109.5	C17—C16—H16A	119.7
H1A—C1—H1B	109.5	C18—C17—C16	119.8 (3)
C4—C1—H1C	109.5	C18—C17—H17A	120.1
H1A—C1—H1C	109.5	C16—C17—H17A	120.1
H1B—C1—H1C	109.5	C17—C18—C19	120.6 (3)
C4—C2—H2A	109.5	C17—C18—H18A	119.7
C4—C2—H2B	109.5	C19—C18—H18A	119.7
H2A—C2—H2B	109.5	C20—C19—C18	119.2 (3)
C4—C2—H2C	109.5	C20—C19—H19A	120.4
H2A—C2—H2C	109.5	C18—C19—H19A	120.4
H2B—C2—H2C	109.5	C15—C20—C19	120.7 (3)
C4—C3—H3A	109.5	C15—C20—H20A	119.6
C4—C3—H3B	109.5	C19—C20—H20A	119.6
H3A—C3—H3B	109.5	C25—C21—C22	102.8 (3)
C4—C3—H3C	109.5	C25—C21—C12	115.3 (2)
H3A—C3—H3C	109.5	C22—C21—C12	111.4 (2)
H3B—C3—H3C	109.5	C25—C21—H21A	109.1
O1—C4—C2	109.7 (3)	C22—C21—H21A	109.1
O1—C4—C1	109.3 (3)	C12—C21—H21A	109.1
C2—C4—C1	114.1 (3)	C23—C22—C21	107.5 (3)
O1—C4—C3	101.0 (2)	C23—C22—H22A	110.2
C2—C4—C3	111.3 (3)	C21—C22—H22A	110.2
C1—C4—C3	110.6 (3)	C23—C22—H22B	110.2
O2—C5—O1	127.7 (3)	C21—C22—H22B	110.2
O2—C5—N1	123.1 (3)	H22A—C22—H22B	108.5
O1—C5—N1	109.2 (3)	C24—C23—C22	104.8 (3)
C7—C6—C11	120.4 (3)	C24—C23—H23A	110.8
C7—C6—N1	130.9 (3)	C22—C23—H23A	110.8
C11—C6—N1	108.6 (2)	C24—C23—H23B	110.8
C6—C7—C8	118.1 (3)	C22—C23—H23B	110.8
C6—C7—H7A	120.9	H23A—C23—H23B	108.9
C8—C7—H7A	120.9	O4—C24—C25	125.2 (3)
C9—C8—C7	122.1 (3)	O4—C24—C23	125.2 (3)
C9—C8—H8A	119.0	C25—C24—C23	109.6 (3)
C7—C8—H8A	119.0	C26—C25—C24	119.6 (3)
C8—C9—C10	119.2 (3)	C26—C25—C21	131.5 (3)
C8—C9—H9A	120.4	C24—C25—C21	108.6 (2)
C10—C9—H9A	120.4	C25—C26—C27	128.6 (3)
C11—C10—C9	119.5 (3)	C25—C26—H26A	115.7
C11—C10—H10A	120.2	C27—C26—H26A	115.7
C9—C10—H10A	120.2	C28—C27—C32	118.2 (3)
C10—C11—C6	120.7 (3)	C28—C27—C26	122.8 (3)
C10—C11—C12	128.9 (2)	C32—C27—C26	119.0 (3)
C6—C11—C12	110.3 (3)	C29—C28—C27	120.4 (3)
C11—C12—C13	102.6 (2)	C29—C28—H28A	119.8
C11—C12—C21	111.6 (2)	C27—C28—H28A	119.8
C13—C12—C21	113.7 (2)	C30—C29—C28	121.0 (3)
C11—C12—C14	110.9 (2)	C30—C29—H29A	119.5
C13—C12—C14	106.1 (2)	C28—C29—H29A	119.5
C21—C12—C14	111.5 (2)	C29—C30—C31	118.9 (4)
O3—C13—N1	125.9 (3)	C29—C30—H30A	120.6
O3—C13—C12	126.1 (2)	C31—C30—H30A	120.6
N1—C13—C12	108.0 (2)	C32—C31—C30	120.8 (4)
C15—C14—C12	113.6 (2)	C32—C31—H31A	119.6
C15—C14—H14A	108.9	C30—C31—H31A	119.6
C12—C14—H14A	108.9	C31—C32—C27	120.7 (3)
C15—C14—H14B	108.9	C31—C32—H32A	119.6
C12—C14—H14B	108.9	C27—C32—H32A	119.6
H14A—C14—H14B	107.7		
			
C5—O1—C4—C2	59.8 (4)	C21—C12—C14—C15	−178.5 (3)
C5—O1—C4—C1	−66.0 (4)	C12—C14—C15—C20	93.2 (3)
C5—O1—C4—C3	177.4 (3)	C12—C14—C15—C16	−86.4 (4)
C4—O1—C5—O2	1.7 (6)	C20—C15—C16—C17	−0.5 (5)
C4—O1—C5—N1	−178.0 (3)	C14—C15—C16—C17	179.2 (3)
C13—N1—C5—O2	5.8 (6)	C15—C16—C17—C18	0.8 (5)
C6—N1—C5—O2	−178.5 (4)	C16—C17—C18—C19	−0.2 (5)
C13—N1—C5—O1	−174.5 (3)	C17—C18—C19—C20	−0.7 (5)
C6—N1—C5—O1	1.2 (5)	C16—C15—C20—C19	−0.4 (5)
C13—N1—C6—C7	−174.9 (3)	C14—C15—C20—C19	179.9 (3)
C5—N1—C6—C7	8.9 (6)	C18—C19—C20—C15	1.0 (5)
C13—N1—C6—C11	1.4 (4)	C11—C12—C21—C25	179.9 (2)
C5—N1—C6—C11	−174.7 (3)	C13—C12—C21—C25	64.5 (3)
C11—C6—C7—C8	−0.9 (5)	C14—C12—C21—C25	−55.4 (3)
N1—C6—C7—C8	175.1 (3)	C11—C12—C21—C22	63.3 (3)
C6—C7—C8—C9	−0.2 (5)	C13—C12—C21—C22	−52.1 (4)
C7—C8—C9—C10	0.7 (6)	C14—C12—C21—C22	−172.0 (3)
C8—C9—C10—C11	−0.2 (5)	C25—C21—C22—C23	−25.9 (3)
C9—C10—C11—C6	−0.8 (5)	C12—C21—C22—C23	98.1 (3)
C9—C10—C11—C12	179.1 (3)	C21—C22—C23—C24	23.3 (3)
C7—C6—C11—C10	1.4 (5)	C22—C23—C24—O4	166.2 (3)
N1—C6—C11—C10	−175.4 (3)	C22—C23—C24—C25	−11.2 (3)
C7—C6—C11—C12	−178.5 (3)	O4—C24—C25—C26	−6.9 (5)
N1—C6—C11—C12	4.7 (4)	C23—C24—C25—C26	170.5 (3)
C10—C11—C12—C13	171.8 (3)	O4—C24—C25—C21	177.5 (3)
C6—C11—C12—C13	−8.3 (3)	C23—C24—C25—C21	−5.1 (3)
C10—C11—C12—C21	49.8 (4)	C22—C21—C25—C26	−156.1 (3)
C6—C11—C12—C21	−130.3 (3)	C12—C21—C25—C26	82.6 (4)
C10—C11—C12—C14	−75.3 (4)	C22—C21—C25—C24	18.9 (3)
C6—C11—C12—C14	104.6 (3)	C12—C21—C25—C24	−102.5 (3)
C5—N1—C13—O3	−7.8 (5)	C24—C25—C26—C27	−175.3 (3)
C6—N1—C13—O3	175.8 (3)	C21—C25—C26—C27	−0.8 (5)
C5—N1—C13—C12	169.7 (3)	C25—C26—C27—C28	44.4 (4)
C6—N1—C13—C12	−6.7 (3)	C25—C26—C27—C32	−135.6 (3)
C11—C12—C13—O3	−173.6 (3)	C32—C27—C28—C29	1.1 (4)
C21—C12—C13—O3	−53.0 (4)	C26—C27—C28—C29	−178.9 (3)
C14—C12—C13—O3	70.0 (4)	C27—C28—C29—C30	0.8 (4)
C11—C12—C13—N1	9.0 (3)	C28—C29—C30—C31	−1.3 (5)
C21—C12—C13—N1	129.6 (3)	C29—C30—C31—C32	−0.1 (5)
C14—C12—C13—N1	−107.5 (3)	C30—C31—C32—C27	2.0 (4)
C11—C12—C14—C15	−53.5 (3)	C28—C27—C32—C31	−2.5 (4)
C13—C12—C14—C15	57.2 (3)	C26—C27—C32—C31	177.5 (2)

### Hydrogen-bond geometry (Å, °)

**Table:**

*D*—H···*A*	*D*—H	H···*A*	*D*···*A*	*D*—H···*A*
C21—H21A···O4^i^	1.00	2.37	3.295 (4)	153
C28—H28A···O4^i^	0.95	2.47	3.401 (4)	165

Symmetry codes: (i) −*x*, *y*−1/2, −*z*+1/2.
